# Individual Differences in the Relationship Between Attachment and Nomophobia Among College Students: The Mediating Role of Mindfulness

**DOI:** 10.2196/jmir.8847

**Published:** 2017-12-14

**Authors:** Ibrahim Arpaci, Mustafa Baloğlu, Hatice İrem Özteke Kozan, Şahin Kesici

**Affiliations:** ^1^ Department of Computers and Instructional Technologies Faculty of Education Gaziosmanpasa University Tokat Turkey; ^2^ Department of Special Education Faculty of Education Hacettepe University Ankara Turkey; ^3^ Department of Counseling Ahmet Kelesoglu Faculty of Education Necmettin Erbakan University Konya Turkey

**Keywords:** reactive attachment disorder, mindfulness, anxiety, phobic disorders, phobia, smartphone

## Abstract

**Background:**

There is a growing interest in nomophobia, which is defined as the fear of being out of cellular phone contact, or "feelings of discomfort or anxiety experienced by individuals when they are unable to use their mobile phones or utilize the affordances these devices provide”. However, only limited research can be found in terms of its determinants at present. Contemporary literature suggests that the relationships among attachment styles, mindfulness, and nomophobia have not been investigated.

**Objective:**

This study aims to investigate the mediating effect of mindfulness on the relationship between attachment and nomophobia. In addition, the study also focuses on gender differences in attachment, mindfulness, and nomophobia. A theory-based structural model was tested to understand the essentials of the associations between the constructs.

**Methods:**

The Experiences in Close Relationships Scale, Nomophobia Questionnaire, and Mindful Attention Awareness Scale were used to collect data from undergraduate students (N=450; 70.9% women [319/450]; mean age=21.94 years [SD 3.61]). Two measurement models (ie, attachment and mindfulness) and a structural model were specified, estimated, and evaluated.

**Results:**

The structural equation model shows that the positive direct effects of avoidant (.13, *P*=.03) and anxious attachment (.48, *P<*.001) on nomophobia were significant. The negative direct effects of avoidant (−.18, *P*=.01) and anxious attachment (−.33, *P*<.001) on mindfulness were also significant. Moreover, mindfulness has a significant negative effect on nomophobia for women only (−.13, *P*=.03). Finally, the Sobel test showed that the indirect effects of avoidant and anxious attachment on nomophobia via mindfulness were significant (*P*<.001). The direct and indirect effects of anxious attachment, avoidant attachment, and mindfulness altogether accounted for 33% of the total variance in nomophobia. Gender comparison results show that there is a significant difference in attachment based on gender (*F*_2,447_=6.97, *P*=.01, Wilk λ=.97, partial *η*^2^=.03). Women (mean 68.46 [SD 16.96]) scored significantly higher than men (mean 63.59 [SD 15.97]) in anxious attachment (*F*_1_=7.93, *P*=.01, partial *η*^2^=.02). Gender differences in mindfulness were not significant (*F*_4,448_=3.45, *P*=.69). On the other hand, results do show significant gender differences in nomophobia (*F*_4,445_=2.71, *P*=.03, Wilk λ=.98, partial *η*^2^=.02) where women scored significantly higher than men.

**Conclusions:**

In general, individuals who are emotionally more dependent and crave more closeness and attention in the relationship tend to display higher levels of fear or discomfort when they have no access to their mobile phones. However, gender has a differential impact on the relationship between avoidant attachment and nomophobia. This study establishes the impact of mindfulness on nomophobia for women; therefore, future studies should test the effectiveness of mindfulness-based therapy approaches and confirm whether they are effective and efficient. On the basis of significant gender difference in nomophobia and attachment, we conclude that gender should be taken into account in mindfulness-based treatments dealing with nomophobia.

## Introduction

### Nomophobia

Excessive or problematic technology use has been partly explained by attachment theory, which posits that people may cultivate connection to technological devices and may feel uncomfortable upon separation [1]. Among various types of problematic technology use, nomophobia is a relatively novel problem [2,3] or disorder [4], which is defined as the fear of being unable to use one’s mobile device [5] or the “the feelings of discomfort or anxiety experienced by individuals when they are unable to use their mobile phones or utilize the affordances these devices provide” [6]. Lin et al [7] categorize nomophobia as a form of situational phobia and suggest its inclusion into the specific phobia in the *Diagnostic and Statistical Manual of Mental Disorders, Fifth Edition* (DSM-V) as a situational phobia [8,9]. Han et al [10] defined nomophobia as a smartphone separation anxiety and argued that when a user perceives the smartphone as an extended self, he or she is more likely to get attached to the device, which in turn would lead to nomophobia by promoting proximity seeking.

Although there is a growing interest in nomophobia, at present, limited research can be found in terms of its determinants. Nonetheless, some of the technology-related situational, dispositional, and environmental antecedents that may share the similar fundamentals with nomophobia are summarized in Table 1. This review indicates that there is a common pattern of dispositional (eg, impulsivity, self-control, self-esteem, or introversion), situational (eg, age, gender, marital status, or ethnicity), and environmental (eg, Web-based social support, amount of time, or stress) antecedents that are associated with technology-related complications.

### Attachment

Attachment styles may be one of the dispositional antecedents of nomophobia, where attachment is defined as “the over-allocation of cognitive and emotional resources towards a particular object, construct, or idea” [21]. Attachment refers to “the mental representations of the self, partner, and relationships called internal working models,” which incorporate self-perceptions of one’s personal worth of love and care from others and of others’ availability and awareness to one’s needs [22]. Attachment theory has originally hypothesized the predisposition of humans to form and preserve strong emotional bonds with their caregivers [23]. However, later, the likelihood of emotional bondage beyond humans has been suggested, in the forms of obsessive-compulsive disorder [24], compulsive work [25], or various types of addictions [26-28]. Thus, Van Gordon et al [29] conclude that unhealthy attachments to objects, people, or situations may be developed.

The relationship between attachment styles and substance addictions has been documented in the literature [30,31]. The mechanisms underlying substance addictions may be similar for behavioral complications such as addictions or phobias in terms of technology, Internet, or social media. For example, anonymous and interactive communication in social media can reduce the feeling of social isolation for individuals with an insecure attachment [32]. Recently, Eichenberg et al [30] reported that individuals with a tendency for Internet addiction were categorized as insecurely attached, whereas securely attached individuals showed no tendency for Internet addiction.

**Table 1:** Selected situational, dispositional, and environmental antecedents of technology-related complications.

Study	Domain	Antecedents
Arpaci et al [11]	Internet addiction	Individualism and psychological needs (ie, achievement, affiliation, dominance, and autonomy)
Karacic and Oreskovic [12]	Internet addiction	Age and gender
Lee et al [13]	Smartphone addiction	Self-expressive and utilitarian benefits and locus of control
Kim and Kang [14]	Mobile messenger services addiction	Self-esteem and self-control
Shen et al [15]	Web-based game addiction	Loneliness, achievement, and excitement
Chen et al [16]	Smartphone addiction	Perceived enjoyment, mood regulation, pastime, and conformity
Jeong et al [17]	Digital game addiction	Loneliness, depression, and aggression
Yang et al [18]	Mobile social networking sites addiction	Web-based social interaction ties and Web-based social supports
Bock et al [19]	Mobile phone affinity	Anxious attachment and addiction correlates with depression and impulsivity
Kim et al [20]	Smartphone addiction	Impulsivity

Studies such as these suggest that attachment theory may help us understand the antecedents of technology-relevant behavioral complications such as nomophobia.

Mindfulness is described as “the awareness that emerges through paying non-judgmental attention on purpose, in the present moment, to the unfolding of experience moment by moment” [33]. Mindfulness is found to have positive effects on physical and psychological well-being [34]. In addition, mindfulness has been theorized to have effects on behavioral addictions, depression, mood disorder, and anxiety disorder [34,35]. Consequently, studies have found mindfulness mediation interventions to improve a variety of health conditions [36].

In sum, an evaluation of the contemporary literature suggests that the relationships among attachment styles and problematic technology use have not been fully investigated [37]. An investigation of the effects of various attachment styles (ie, avoidant attachment vs anxious attachment) on nomophobia would be highly original in the literature. Furthermore, studying the potential effects of mindfulness on the association between attachment and nomophobia would be interesting and theoretically warranted. Lastly, because gender roles theory posits that there exist cognitive and emotional differences between men and women, this research aimed to investigate the effects of gender differences on attachment, mindfulness, and nomophobia.

### Theoretical Background and Hypothesis

#### Attachment Theory

Bowlby’s [22,38-43] classical research on the infant-mother attachment theory has been extended to different populations [36,37], relationship types [38,39], and nonhuman objects [28,44]. Hazan and Shaver [45] classified attachment styles into three general categories, that is, secure, anxious, and avoidant, which are consistent with the three childhood attachment styles suggested in the original studies with infants.

*Secure attachment* is associated with a positive model of self as well as a positive model of others. Therefore, individuals who score lower on both dimensions are classified as *insecurely* attached [46]. *Avoidant attachment* is associated with a positive model of self and a negative model of others. It can be construed as hypersensitivity to unresponsiveness, rejection, and abandonment. Individuals whose dominant style is avoidant tend to have difficulties with close relationships and intimacy and seek to maintain self-reliance, emotional distance, and control [45]. They distance themselves from others and avoid experiencing negative emotions [47].

*Anxious attachment* is associated with a negative model of self and a positive model of others [45,46]. Anxious attachment is characterized by greater emotional dependence, desire for more commitment and closeness [48], and more intensive attention from partners [47]. Individuals dominant in this attachment style tend to have a fear of rejection, a negative perception of self-worth, worry about being abandoned or unloved, and thereby, seek a higher need for closeness and intimacy [49]. They seem to be obsessive and hypervigilant in the relationship [50]. This study opted to focus on the effects of anxious versus avoidant attachment styles on nomophobia and mindfulness.

Attachment styles have an impact on social interactions and emotional development [51,52], which in turn determine the risk of developing dependence to people, objects, or events [26,53]. Recent studies indicate a significant relationship between attachment styles (ie, avoidant vs anxious) and behavioral addictions [26-28]. In addition, attachment styles are related to the problematic use of technology such as the Internet [54], mobile phones [28], video games [55], and social media [26]. More specifically, anxious attachment was associated with technology-mediated breakups, whereas avoidant attachment predicted the likelihood of technology overuse [56]. Blackwell et al [26] found that both attachment styles predict social media addiction. On the basis of attachment theory and available research findings, we hypothesized that the effects of both anxious attachment (hypothesis 1) and avoidant attachment (hypothesis 2) on nomophobia would be *positively* significant.

#### Mindfulness

Mindfulness-based mental health betterment techniques assert that behavioral tendencies that lead to psychological complications can distort individuals’ perceptions of reality and may cause maladjustment [57]. Accordingly, the American Psychiatric Association [58] promotes mindfulness-based therapy approaches, which have been found effective in the treatment of behavioral addictions, depression, mood disorder, and anxiety disorder [35,59].

There is promising evidence for the efficacy of mindfulness-based treatment, suggesting that self-awareness increased through mindfulness practices can target multiple neural, psychological, physiological, and behavioral processes [34,35,60-63]. In the same vein, recent studies argued that mindfulness-based interventions could treat behavioral complications such as Internet addiction [64] and video game addiction [65]. Problematic technology use, such as nomophobia, may also be treated by interventions that gear toward enhancing individuals’ mindfulness levels [66]. Therefore, we hypothesized that higher levels of mindfulness would be associated with lower levels of nomophobia. In other words, there would be a negatively significant effect of mindfulness on nomophobia (hypothesis 3).

There is strong evidence that mindfulness is associated with attachment styles. For example, Pepping et al [67] indicated that avoidant and anxious attachment were significantly associated with lower scores in mindfulness. In another study, mindfulness was negatively linked with anxious attachment [68]. Furthermore, studies have validated the association between mindfulness and attachment styles [69-71]. On the other hand, several studies argued that mindfulness is significantly associated with attachment security [67,72]. Thus, we hypothesized that there would be a significant negative effect of both anxious attachment (hypothesis 4) and avoidant attachment (hypothesis 5) on mindfulness. Finally, based on gender roles theory, we expected significant gender differences between men and women in attachment, mindfulness, and nomophobia.

## Methods

### Participants

A total of 450 students were recruited through convenience sampling method. Of the 450 students, 319 were women (70.9%) and 131 were men (29.1%). Participants’ ages ranged from 18 to 40 years (mean age=21.94 [SD 3.61]). In terms of ownership, 99.3% of the participants had a smartphone (447/450), whereas 95.6% (430/450) had mobile Internet. Participants used mobile Internet on an average of 4.58 hours a day (SD 2.92). In terms of college levels, 123 students were freshmen (27.3%); 11 were sophomores (2.4%); 153 were juniors (34.0%); and 163 were seniors (36.3%). Participants came from various study majors such as Psychology (41.3%), Social Science (33%), Health Science (17.1%), and Computer Science (8.6%).

### Measures

#### The Experiences in Close Relationships (ECR) Scale

Brennan et al [46] developed the ECR scale, a 36-item, self-report attachment measure, which includes 2 subscales: Avoidance (alpha=.94) and Anxiety (alpha=.91). Sample items from the scale include “I prefer not to show a partner how I feel deep down” (ie, Avoidance) and “worry about being abandoned” (ie, Anxiety). Items are rated on a 7-point Likert scale, ranging from 1 (strongly disagree) to 7 (strongly agree), and higher scores refer to higher levels of avoidance or anxiety. Sümer [73] provided evidence for the reliability and validity of the Turkish version of the ECR scale. Cronbach alpha coefficients for anxiety and avoidance styles were found to be .86 and .90, respectively [73]. In this study, the instrument’s internal consistency coefficients for the combined groups, men, and women were found to be acceptable (Table 2).

#### The Mindful Attention Awareness Scale (MAAS)

Brown and Ryan [74] developed 15 items rated on a 6-point Likert scale that form a single factor structure to assess the awareness of the present moment and the level of attention. Sample items from the scale include “I could be experiencing some emotion and not be conscious of it until sometime later” and “I find it difficult to stay focused on what’s happening in the present.” Özyeşil et al [75] adapted the scale into Turkish and provided validity and reliability properties of the adapted scale. The scale’s internal consistency coefficient was found to be .85 in this study (Table 2).

#### The Nomophobia Questionnaire (NMP-Q)

The NMP-Q is a 20-item, 5-point Likert type, self-report instrument that assesses the fear of being unable to use mobile devices under 4 subscales (ie, Unable to Access Information, Losing Connectedness, Unable to Communicate, and Giving Up Convenience). Sample items from the questionnaire include the following: “I would be annoyed if I could not look information up on my smartphone when I wanted to do so” (ie, Unable to Access Information), “If I could not use my smartphone, I would be afraid of getting stranded somewhere” (ie, Losing Connectedness), “I would be anxious because I could not keep in touch with my family and/or friends” (ie, Unable to Communicate), or “I would feel awkward because I could not check my notifications for updates from my connections and Web-based networks” (ie, Giving Up Convenience) [5]. Cronbach alpha of the original scale was .92. Yildirim et al [76] provide the validity and reliability evidence of the Turkish NMP-Q. They found that Cronbach alpha coefficients of the 4 subscales were .90, .74, .94, and .91, respectively. We used the total nomophobia scores in this study, which are computed by summing the 4 subscale scores. The internal consistency coefficients of the questionnaire ranged from .89 to .92 for the combined groups, men, and women in this study (Table 2).

### Procedure

All procedures were performed in accordance with the ethical standards of the institutional board guidelines, and the study was approved by the institutional review board. Before informed consent was obtained, participants were debriefed about the nature and possible consequences of the study. Participation was voluntary and participants received extra course credit for completing the research packet.

**Table 2:** Descriptive statistics, correlation coefficients, convergent validity and discriminant validity, and reliability of the study variables. Statistics reported outside parentheses are for the combined groups, whereas within parentheses are statistics for men and women, respectively.

Variables	Alpha	CR^a^	AVE^b^	Correlations			
				1	2	3	4
1. Anxious	.88 (.87-.89)	.77 (.80-.76)	.52 (.50-.50)	*.72* (.71-.71)^c^			
2. Avoidant	.77 (.75-.77)	.84 (.83-.83)	.57 (.55-.55)	.09 (.18-.10)	*.75* (.74-.74)		
3. Mindfulness	.85 (.87-.85)	.79 (.70-.78)	.50 (.50-.51)	−.26 (−.24 to −.25)	−.22 (−.42 to −.15)	*.71* (.71-.71)		
4. Nomophobia	.91 (.89-.92)	.75 (.81-.77)	.50 (.50-.50)	.54 (.47-.56)	.27 (.23-.28)	−.35 (−.35 to −.35)	*.71* (.71-.71)
Range				1-7	1-7	1-6	1-5
Mean				4.31 (4.47-4.24)	3.82 (3.55-3.93)	3.77 (3.75-3.78)	3.25 (3.22-3.26)
SD^d^				1.75 (1.70-1.75)	1.73 (1.63-1.74)	1.33 (1.38-1.31)	1.10 (1.11-1.09)

^a^CR: composite reliability.

^b^AVE: average variance extracted (for convergent validity).

^c^Discriminant validity coefficients are on the diagonal.

^d^SD: standard deviation.

## Results

### Instrument Reliability and Validity

The normality of the scales suggested minimal skewness (range −.34 to .33) and kurtosis (range −.50 to 2.11). Convergent and discriminant validity of the constructs were investigated by developing a correlation matrix. Hair et al [77] suggest that convergent validity is adequate when average variance extracted (AVE) coefficients exceed or equal .50. In this study, the square roots of the AVE coefficients (shown in diagonal Table 2) were greater than the interconstruct correlations for all constructs. Thus, discriminant validity was found satisfactory for the constructs studied. Table 2 also illustrates reliability and convergent validity coefficients along with descriptive statistics for the combined groups, women, and men.

### Gender Differences

One-way multivariate analysis of variances investigated statistical differences between men (n=131) and women (n=319) in attachment, mindfulness, and nomophobia. Results show that there is a significant difference in attachment based on gender (*F*_2,447_=6.97, *P*=.01, Wilk λ=.97, partial *η*^2^=.03). Women (mean 68.46 [SD 16.96]) scored significantly higher than men (mean 63.59 [SD 15.97]) in anxious attachment (*F*_1_=7.93, *P*=.01, partial *η*^2^=.017). Gender differences in mindfulness were not statistically significant (*F*_4,448_=3.45, *P*=.69). On the other hand, results show significant gender differences in nomophobia (*F*_4,445_=2.71, *P*=.03, Wilk λ=.98, partial *η*^2^=.02), where women scored significantly higher than men.

### Confirmatory Factor Analysis

Confirmatory factor analysis (CFA) was used to test whether the proposed measurement models and the structural model were to fit the data. The model fit indices for the measurement models and the structural model are presented in Table 3. Results suggest that the measures used in this study formed adequate measurement models and therefore, provided evidence for the construct validity.

### Hypothesis Testing

**Figure 1:**
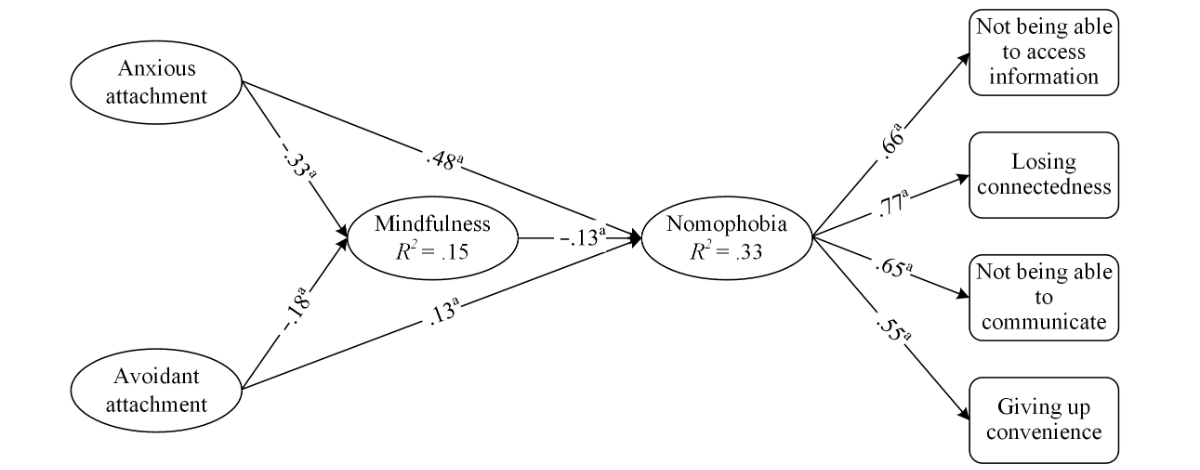
Hypothesis testing results. Manifest variables and their respected error terms underlying the latent variables are omitted to save space. "a" signifies *P*<.001.

To test the research model and hypotheses, structural equation modeling (SEM) was employed with maximum likelihood estimation. The SEM results for the combined groups, men, and women are presented in Table 4. As shown in the table, the positive direct effect of anxious attachment was significant on nomophobia for the combined groups, women, and men; however, the direct effect of avoidant attachment on nomophobia was significant only for the combined groups but not for men or women. Therefore, results shown in Figure 1 supported hypothesis 1 but not hypothesis 2. Moreover, there is a significant negative effect of mindfulness on nomophobia for the combined groups (−.13, *t*_419_=−2.16, *R*^2^=.15, *P=*.03) and women (−.15, *t*_318_=−2.23, *R*^2^=.15, *P=*.03) but not for men (−.13, *t*_130_=−1.12, *P=*.26). Thus, results supported hypothesis 3 for the combined groups and women but not for men.

**Table 3:** Fit indices of the measurement models and the theoretical model. The results of multigroup analysis are shown in parentheses.

Fit indices	Attachment	Nomophobia	Structural model	Acceptable values
Chi-square	83.51 (134.12)	401.75 (574.95)	333.14 (612.78)	
Degrees of freedom	33 (66)	156 (310)	216 (432)	
*P* value	.001 (.001)	.001 (.001)	.001 (.001)	.05≤ *P* ≤1.00 [79]
Chi-square/degrees of freedom	2.53 (2.03)	2.58 (1.86)	1.54 (1.42)	<3 [78
GFI^a^	.97 (.94)	.92 (.89)	.94 (.90)	≥.90 [78,79]
AGFI^b^	.94 (.91)	.89 (.86)	.92 (.87)	≥.80 [78,79]
RMR^c^	.03 (.03)	.06 (.07)	.02 (.03)	<.05 [78,79]
RMSEA^d^	.06 (05)	.06 (.04)	.04 (.03)	<.08 [77]	
NFI^e^	.94 (.90)	.92 (.89)	.90 (.81)	≥.90 [77]
TLI^f^	.95 (.93)	.93 (.93)	.95 (.92)	≥.90 [78,79]
CFI^g^	.96 (.95)	.95 (.94)	.96 (.94)	≥.90 [78,79]
IFI^h^	.96 (.95)	.95 (.94)	.96 (.94)	≥.90 [78,79]

^a^GFI: Goodness-of-Fit Index.

^b^AGFI: Adjusted Goodness-of-Fit Index.

^c^RMR **:** root mean square residual.

^d^RMSEA: root mean square error of approximation.

^e^NFI: Normed Fit Index.

^f^TLI: Tucker-Lewis Index.

^g^CFI: Comparative Fit Index.

^h^IFI: Incremental Fit Index.

**Table 4:** Structural equation modeling (SEM) and multigroup analysis results. Results are reported for the combined groups (men, women), respectively.

Hypothesis	Estimate	SE^a^	Critical ratio	*P* value
Anxious → Nomophobia	.48 (.53-.46)	.17 (.42-.18)	6.93 (3.53-5.82)	.001 (.001-.001)	
Avoidant → Nomophobia	.13 (.02-.12)	.30 (.37-.29)	2.21 (.18-1.81)	.03 (.86-.07)
Mindfulness → Nomophobia	−.13 (−.13 to −.15)	.35 (.63-.44)	−2.16 (−1.12 to −2.23)	.03 (.26-.03)
Anxious → Mindfulness	−.33 (−.26 to −.31)	.03 (.06-.03)	−4.66 (−2.19 to −3.75)	.001 (.03-.001)
Avoidant → Mindfulness	−.18 (−.33 to −.15)	.06 (.09-.05)	−2.63 (−2.28 to −2.05)	.01 (.02-.04)

^a^SE: standard error.

Furthermore, negative direct effects of both anxious and avoidant attachment on mindfulness were significant for the combined groups, men, and women. Therefore, results supported both hypothesis 4 and hypothesis 5. The direct and indirect effects of anxious attachment, avoidant attachment, and mindfulness altogether accounted for 33% of the total variance in nomophobia. The equations shown in Table 5 represent the relationships among the constructs.

### Mediation Analysis

A 4-step approach was used to test the mediation effect of mindfulness on the relationship between attachment and nomophobia. First, the direct effects of both anxious (SE=.13, *t*=−3.19, *P*<.001) and avoidant (SE=.18, *t*=−3.54, *P*<.001) attachment on mindfulness were found significant. Second, both the direct effects of anxious (SE=.41, *t*=4.02, *P*<.001) and avoidant (SE=.39, *t*=2.11, *P=*.05) attachment on nomophobia were found significant as well. Third, the direct effect of mindfulness on nomophobia was significant (SE=.65, *t*=−3.50, *P*<.001). Finally, the Sobel test showed that the indirect effect of avoidant and anxious attachment on nomophobia via the mediator (ie, mindfulness) was significant (*P*<.001). These results supported full mediation and indicated that attachments have significant effects on nomophobia through mindfulness.

### Randomization Tests

**Figure 2:**
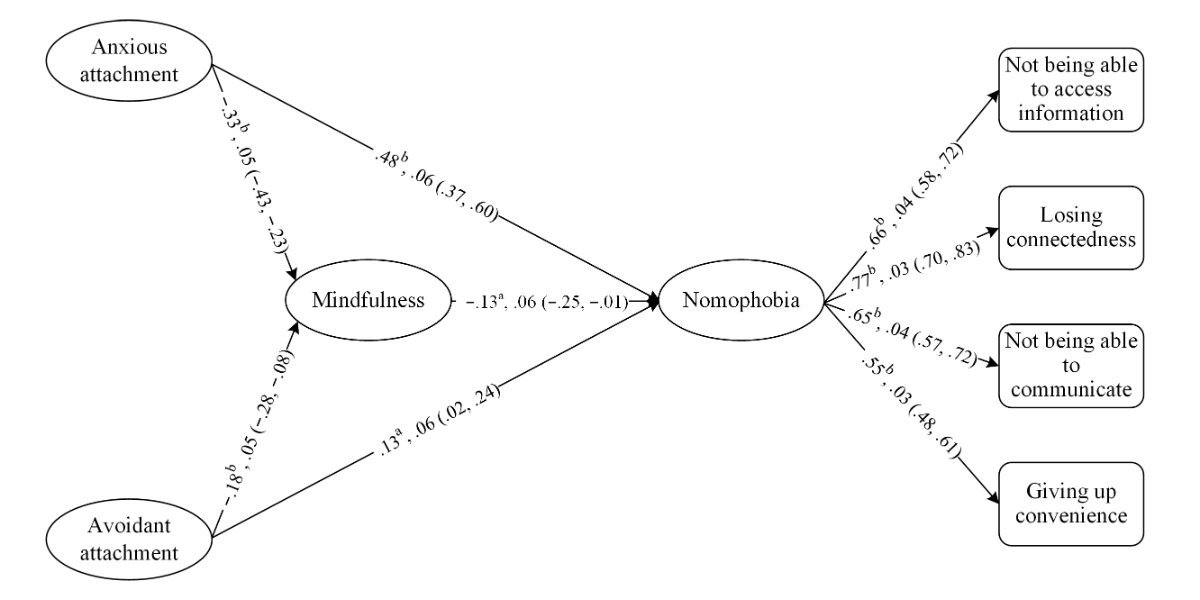
Means of 5000 bootstrap replicate coefficients and their standard errors. Within parentheses are 95% CIs. Manifest variables and their respected error terms underlying the latent variables are omitted to save space. "a" signifies *P*=.05 and "b" signifies *P*<.001.

Because we used a nonrandom sample, randomization tests were conducted to support generalizability beyond this study’s sample. We employed 5000 bootstrap replicates to test the effects of attachment on nomophobia via mindfulness. Means, standard errors, 95% CIs, significance levels, and the directions of the relations are reported in Figure 2, which altogether suggest that similar results closely approximated in the bootstrapped samples.

**Table 5:** Relationships among the constructs. Results are reported for the combined groups (men, women), respectively.

Variable	Equation	Error	*R*^2^
Mindfulness	−.18 (−.33 to −.15) x Avoidant −.33 (−.26 to −.31) x Anxious	.13 (.15-.13)	.15 (.24-.12)
Nomophobia	.13 (.02-.12) x Avoidant .48 (.53-.46) x Anxious −.13 (−.13 to −.15) x Mindfulness	3.83 (3.55-3.94)	.33 (.36-.31)

## Discussion

### Principal Findings

One way of explaining why people experience problems in technology use is that of attachment theory. Recent research has found significant associations among different attachment styles and problematic use of the Internet, mobile phones, video games, and social media [26-28]. Therefore, the aim of this study was to further investigate the effects of anxious and avoidant attachment on nomophobia. Additionally, because the concept of mindfulness has been theorized to have plausible effects on psychological complications, we aimed to test whether it would mediate the association between attachment and nomophobia.

In general, we found positive effects of both anxious and avoidant attachment on nomophobia and their negative effects on mindfulness. However, when we analyzed the details of the effects for men and women separately, results revealed interesting patterns. For example, anxious attachment had a significant positive effect on nomophobia for both women and men, even though the link was stronger among men than women. Individuals who are emotionally more dependent and crave for more closeness and attention in the relationship tend to display higher levels of fear or discomfort when they have no access to their mobile phones. Thus, we conclude that anxious attachment is reflected upon a technological object, that is, a smartphone. These findings suggest the likelihood of a strong link between anxious attachment and nomophobia, which should be further scrutinized through experimental studies.

When it comes to avoidant attachment, results are not as straightforward. We found that avoidant attachment shows a significant positive effect on nomophobia on the combined groups (N=450); however, the effects reduced to nonsignificance when men (n=131) and women (n=319) were analyzed separately. This suggests that gender has a differential impact on the relation between avoidant attachment and nomophobia.

Literature suggest that mindfulness cultivates a better understanding of the nature of existence by mindfully observing the dissolution of affective, cognitive, and sensory processes [29]. In addition, mindfulness is used effectively in various behavioral dependencies such as Internet gaming disorder [35,80]. Lastly, it has been suggested that technology-based addictions may be treated by interventions that gear toward enhancing mindfulness [34,35,61,81]. This study is the first attempt in the literature to investigate the effect of mindfulness on nomophobia. Results indicate a significant direct effect of mindfulness on nomophobia for women but not for men. Women who show higher levels of mindfulness carry lower risk of nomophobia. Similar to our results, Sriwilai and Charoensukmongkol [37] examined the relationship between social media addiction and mindfulness. They found that individuals with higher social media addiction have lower mindfulness scores. These findings indicate that mindfulness-based therapies could be used in the treatment of behavioral addictions for women. Similar suggestions were made by Wahbeh and Oken [36], Garland et al [82], Black [83], Peltz and Black [84], and Garland [85].

Prior studies suggested that men and women show quite different Web-based behavior patterns. For example, Durkee et al [86] indicated that male adolescents prefer to use Web-based games, whereas female adolescents prefer to interact with social networking websites. Our findings broaden the literature by suggesting the possibility of using mindfulness-based treatment techniques with nomophobia for women. There are two general implications of these findings. First, the concept and techniques of mindfulness can be used as a preventive tool to dodge the risk of nomophobia for women. Once risk assessments are completed and under-risk groups are identified, mindfulness exercises may be taught and practiced with under-risk women. Second, mindfulness-based treatment techniques may be used with clinically diagnosed women to remedy the higher levels of nomophobia. This study establishes the impact of mindfulness on nomophobia at least for women; therefore, future studies should test the effectiveness of mindfulness-based therapy approaches and confirm whether they are effective and efficient.

Findings indicate that there is a significant difference in nomophobia between women and men. Furthermore, there was a significant difference in the attachment styles based on gender. Results indicate that women and men differed in anxious attachment but not in avoidant attachment. These findings imply that women tend to display more anxious attachment than men. Gender differences in attachment can be explained by gender schema theory proposed by Bem [87]. She theorizes that individuals develop cognitive schemas throughout childhood to be able to show gender-appropriate behaviors. Such schemas eventually predispose men and women to form and preserve emotional bonds differently. Previously, Katz and Toner [88] conducted a systematic review to figure out the role of gender differences in the effectiveness of the mindfulness-based treatment for substance use. Their findings suggested that men gravitate less toward the treatments, and thereby, women benefit more from such treatments. These findings imply that gender differences should be considered in mindfulness-based treatments dealing with nomophobia.

### Limitations

There are several limitations of this study. First, using a convenience sampling in recruiting the participants is one of the limitations of the study. We attempted to overcome this limitation by conducting randomization tests; however, future studies may analyze the hypothesized relationships on longitudinal data or random samples. Second, the study shows an overwhelming use of female subjects as compared with male subjects; the sample size for men is quite limited to test SEM-based analyses, and we suggest that the models established in this study be tested with a larger sample, which is fairly well balanced by gender. Third, neither is the attachment style the only determinant of nomophobia nor is mindfulness the only mediator; however, based on cyber psychology literature, the study focused on these factors. Other dispositional or situational factors (ie, personality characteristics) and mediators (ie, psychological needs) would definitely be worthwhile to explore in future studies. Finally, it would be useful to incorporate qualitative techniques for a detailed investigation of the research problem. Despite these limitations, this study has significant contributions to expand the literature by identifying important relationships among attachment, mindfulness, and nomophobia.

### Conclusions

We found the significant effects of both anxious and avoidant attachment on mindfulness and nomophobia. However, gender has a differential impact on the relation between avoidant attachment and nomophobia. We conclude that gender differences should be considered in mindfulness-based treatments dealing with nomophobia. Mindfulness-based therapies could be used in the treatment of nomophobia, especially for women.

## Abbreviations

AGFI: Adjusted Goodness-of-Fit Index
AVE: average variance extracted
CFA: confirmatory factor analysis
CFI: Comparative Fit Index
CR: composite reliability
ECR: Experiences in Close Relationships
GFI: Goodness-of-Fit Index
IFI: Incremental Fit Index
MAAS: Mindful Attention Awareness Scale
NFI: Normed Fit Index
NMP-Q: Nomophobia Questionnaire
RMR: root mean square residual
RMSEA: root mean square error of approximation
SD: standard deviation
SE: standard error
SEM: structural equation modeling
TLI: Tucker-Lewis Index
